# The Wisconsin Society of Veterinary Graduates

**Published:** 1900-03

**Authors:** W. G. Clark

**Affiliations:** Secretary


Secretary.

### THE WISCONSIN SOCIETY OF VETERINARY GRADUATES.

THE semi-annual meeting was held at the Kirby House, Milwaukee, September 13, 1899. The meeting was called to order by the President, Dr. Roberts, at 2 P.M., in the parlors of the Kirby House. Present: Drs. H. Arpke, W. G. Clark, C. Evans, E. R. Flack, C. H. Ormond, R. Kuoni, G. Ed. Leech, E. L. Morgenroth, E. H. Newton, J. F. Roub, D. Roberts, S. S. Snyder, and L. A. Wright. Visitors: G. W. Butler, Milwaukee; E. A. Manuel, Desplaines, Ill.; A. R. Wake, Cudahy; Jos. Pfieffer, Sheboygan; W. E. A. Wyman, Milwaukee; Wm. Voss, Kiel; H. F. Eckert, Markesan; P. J. Wilkinson, Oconomowoc; S. J. Collins, Reedsburg, and E. A. McCullough, Delevan.

The minutes of the last meeting were read and approved. The following applications for membership were received: Drs. W. E. A. Wyman, Wm. Voss, H. F. Eckert, P. J. Wilkinson, and S. J. Collins.

In the absence of the censors the President appointed as a commitee to examine the applications Drs. Roub, Clark, and Ormond. A recess was taken until the committee reported on the applications. After the meeting was called to order the committee reported favorably on the applications.

On motion, the candidates were voted on by acclamation. The vote was taken and the several gentlemen were declared elected members of the Society.

Dr. Leech reported on behalf of the Committee on Legislation in regard to the law passed at the last session of the Legislature. The question of prosecution of non-graduates under the new law was discussed by Drs. Leech, Roub, Ormond, Flack, Wright, and Roberts.

Dr. Hartwig not being present, his paper on "Parturient Apoplexy" was read by the Secretary. Discussed by Drs. Wright, Roub, Wyman, Clark, Flack, Arpke, Collins, Morgenroth, Kuoni, and Snyder.

On motion, the Society adjourned to meet at 8 P.M.

*Evening Session.* The meeting was called to order by the President at 8 P.M. In addition to those present at the afternoon session were Bureau of Animal Industry Inspectors Drs. A. E. Behnke, H. Caldwell, W. J. Stewart, and R. H. Harrison. Several of the leading horseshoers of the city were also present.

The meeting was opened under the head of applications for membership, and the application of Dr. E. A. McCullough was received. The application being reported on favorably by the censors, it was balloted on, and Dr. McCullough was declared elected to membership.

Dr. L. A. Wright read a very able paper on the "Workings of Heredity." Discussed by Drs. Roub, Schmitt, Leech, Flack, Wyman, and Butler. On motion, the discussion was closed and the essayist excused.

Dr. E. L. Morgenroth gave a short talk on shoeing and exhibited several shoes. Discussed by Drs. Arpke, Schmitt, Roub, Wright, and Leech, also by Messrs. Widemeyer and Dorman, who gave us some very good ideas in regard to shoeing. On motion, the discussion was closed and the essayist excused. On motion, the Society adjourned to meet in Madison, subject to the call of the President and Secretary.

Thursday morning at 8.30 the Society met at Dr. W. E. A. Wyman's hospital. Dr. J. F. Roub performed a skilful and successful operation on a ridgling and explained his method of operating. Dr. Wyman performed cunean tenectomy for bone spavin.

W. G. CLARK,
Secretary.

